# The effects of multifactorial pharmacist-led intervention protocol on medication optimisation and adherence among patients with type 2 diabetes: A randomised control trial

**DOI:** 10.12688/f1000research.146517.2

**Published:** 2024-09-16

**Authors:** Marwan El-Deyarbi, Luai Ahmed, Jeffrey King, Syed Abubackar, Ahmed Al Juboori, Nirmin A. Mansour, Salahdein Aburuz

**Affiliations:** 1 Department of Pharmacy, Oud Al-Touba Diagnostic and Screening Clinic, Ambulatory Health Services, Abu Dhabi Health Services Co. SEHA, Al Ain, Abu Dhabi, 00971, United Arab Emirates; 2 Department of Pharmacology, College of Medicine and Health Sciences, United Arab Emirates University, Al Ain, Abu Dhabi, 00971, United Arab Emirates; 3 Institute of Public Health, College of Medicine and Health Sciences, United Arab Emirates University, Al Ain, Abu Dhabi, 00971, United Arab Emirates; 4 Department of Veterans Affairs, Department of Geriatrics and Extended Care, Greater Los Angeles Veterans Research and Education Foundation, Los Angeles, California, USA; 5 David Geffen School of Medicine, University of California Los Angeles, Los Angeles, California, USA; 6 Division of Endocrinology, Oud Al-Touba Diagnostic and Screening Clinic, Ambulatory Health Services, Abu Dhabi Health Services Co. SEHA, Al Ain, Abu Dhabi, 00971, United Arab Emirates

**Keywords:** Diabetes, medication adherence, pharmacist-led, fixed medication possession ratio, medication therapy management, mobile application

## Abstract

**Background:**

Patient-related factors and limited medication adherence in patients with chronic diseases, are associated with poor clinical outcomes, long-term complications, and increased overall disease costs. Many methods have been tested with mixed results, and innovative approaches are needed to encourage patients to adhere to their prescribed drug regimens.

**Methods:**

This randomised controlled trial examined a new multifactorial pharmacist-led intervention protocol (MPIP), including a medication therapy management (MTM) program with face-to-face counselling, patient-specific medication booklets, and a mobile application, from July 2021 to September 2022 in the Oud Al Touba diagnostic and screening ambulatory centre in 192 patients with type 2 diabetes in the United Arab Emirates. Medication adherence was assessed using the fixed medication possession ratio of medication refills and the medication adherence questionnaire.

**Results:**

At 12 months follow-up, participants in the MPIP showed significant improvement in overall medication adherence with total (composite) medication possession ratio (MPRt) of mean (±SD) 0.95 (±0.09) compared to 0.92 (± 0.09) in the control group with mean difference of 0.03 (95%, CI 0.01–0.06), P =0.02. In addition, improvement trend was evident in the MPIP group for all medication regimens with P value <0.01. Comparable results were noticeable in adherence questionnaire scores at the end of the study, with 66 participants in the intervention group scored zero on the questionnaire, suggesting high adherence to medication compared to the control group (48 participants only). The MTM program performed 41 clinical interventions on drug-related problems, compared to six interventions in the control group, and the use of mobile application and medication booklet have increased to 45.7% compared to 21.4% before study exit.

**Conclusions:**

The pharmacy intervention protocol effectively improved medication adherence and optimised medication regimens in diabetic patients with chronic medication regimens in an ambulatory healthcare centre.

## 1. Introduction

In 2021, the United Arab Emirates (UAE) had the highest worldwide prevalence of diabetes in the world, with approximately 12.3% of its population diagnosed with diabetes, and over 90% of cases of diabetes are caused by type 2 diabetes. ^1^ This high prevalence is accompanied by high rates of other risk factors for chronic kidney disease, ^2^ and cardiovascular disease, including obesity in 35%, dyslipidaemia in 44%, and hypertension in 23.1% of the population in Abu Dhabi. ^3^

Several studies showed that suboptimal duration of medication use (non-persistence with medication) in chronic diseases such as type 2 diabetes is attributed to poor clinical outcomes, including higher glycated haemoglobin (HbA1c) and diabetic complications including diabetic nephropathy and mortality. ^4^^,^^5^

Moreover, the estimated annual cost savings for individuals achieving optimal medication adherence with medication possession ratio (MPR) ≥ 0.6 from less than 0.6, is around 661 million dollars in the United States of America and may reach 1.16 billion dollars if we succeed in approaching MPR = 1 for medication non-adherence patients. This cost saving can be explained by higher inpatient and emergency room visit costs which would exceed any savings from less intensive ambulatory visits and outpatient medication costs. ^6^

In addition, studies have shown that patient-related factors such as age, personal beliefs or educational status, are the most influential factors in medication adherence rather than medication cost or insurance-related payment. ^7^ Moreover, medication adherence information is inaccurately represented in patients’ electronic medical records. ^8^ These elements raise the need for innovative patient-centred tools to enhance proper medication adherence, documentation, and tracking. ^9^

Moreover, medication-related factors such as medication class and side effects are major contributors to non-adherence. ^10^ Medication side effects or drug-drug interactions may lead to safety or tolerability issues which negatively affect the overall patient’s clinical outcomes and quality of care. Most of these factors have a long-term effect on diabetes and hard endpoints such as cardiovascular events and all-cause mortality. ^11^

Evidence from a systematic review and meta-analysis showed that individual pharmacist-led interventions, such as face-to-face interventions, are beneficial in improving medication adherence and patient outcomes and showed consuming 84% of the prescribed doses in the treatment group compared to 77% only of prescribed doses in the control group. ^12^ Another meta-analysis showed that pharmacist interventions, whether carried out independently or in coordination with other medical specialists, can improve medication adherence and patients’ clinical outcomes such as blood pressure (BP) reduction in both systolic and diastolic BP (-7.6 and -3.9 mmHg, respectively) with a more larger effect if the intervention carried out at least monthly. ^13^

The use of technology such as mobile applications and telemedicine has proven to be beneficial for chronic disease management. ^14^ Studies have shown the effectiveness of the telemedicine system approach on overall healthcare quality and that it can be complementary to conventional face-to-face monitoring of diabetic patients. This can be a useful tool for the treatment of complex patients who require more frequent visits to achieve tighter glycaemic control or have difficulty accessing the healthcare system ^15^; however, few studies have investigated the correlation between medication counselling through telemedicine and patient adherence and the long-term effects on diabetes or related complications especially in the Middle East.

The Emirates of Abu Dhabi holds the largest integrated hospital and ambulatory pharmacy network in the UAE and a patient’s electronic medical record (Cerner ^®^) linked to private pharmacies, however, there is no evidence-based process for measuring or improving patients’ medication adherence.

Given the diversity of pharmacist-led interventions as a strategy to improve medication adherence and clinical outcomes, this study was conducted to address the effect of a new multifactorial pharmacist-led intervention protocol (MPIP) which consists of two interrelated strategies: first, thorough medication counselling and reassurance to enhance adherence to prescribed medication through medication adherence possession data, and second, a comprehensive medication therapy management (MTM) with a clinical pharmacist to improve medication management and adherence in the diabetic population in the UAE.

## 2. Aims and objectives

This study aimed to assess the impact of pharmacist-led multifactorial interventions on medication management optimisation and patient adherence to medications.

The primary objectives were as follows: 1) to evaluate the impact of the new protocol (MPIP) on overall medication adherence measured by the total medication possession ratio (MPRt) for all regimens; 2) to measure the difference in self-reported adherence questionnaire from baseline and study exit in both study groups; and 3) to assess the number and type of clinical interventions in the MTM program.

The secondary objectives were as follows: 1) compare the effect of MPIP on regimen-specific MPRs in the intervention group for antihyperglycaemic, antihypertensive, and antihyperlipidaemic regimens at the end of the study with the control group; 2) measure the improvement in regimen non-persistence between the intervention and control groups; and 3) analyse the changes in usage patterns of the mobile application and patient medication booklet and correlate it with patient adherence.

## 3. Methods

### 3.1 Study design and oversight

This was a randomised control trial of 12 months duration, among type 2 diabetic patients recruited from the endocrinology and chronic disease (CDC) outpatient clinics at Oud Al Touba Diagnostic and Screening Centre, Al Ain, UAE.

This article presents one outcome of the original trial that was registered at the US National Institutes of Health (name of registry: ClinicalTrials.gov, registration number: NCT04942119, registration date: 05/21/2021, and URL of trial in the registry database: https://clinicaltrials.gov/study/NCT04942119)), and the original trial was approved by the Abu Dhabi Health Services Company (SEHA) Research Oversight and Ethics Committee (SEHA REC) in the United Arab Emirates on April 2021 (approval number: SEHA-IRB-021) Supplementary File 1 found as *Extended data* ^46^). Written informed consent was obtained from all participants before study recruitment and they received a copy of it along with the study information sheet (Supplementary File 2, 3 found as *Extended data* ^46^). This study follows the CONSORT checklist and flow diagram. ^47^

We included male and female patients between 30 and 65 years of age, previously diagnosed with type 2 diabetes, with unchanged diabetic medication for 4 months prior to study enrollment, and with an estimated glomerular filtration rate (eGFR) above 30 ml/minute/1.73 m ^2^ of body surface area. Participants were excluded if they were refilling their medication from other pharmacies not integrated with Cerner electronic health record, through a home care service, or had physiological barriers as assessed by the clinician that may affect patient interviews and filling the adherence questionnaire.

Simple randomisation was performed using a computer-generated random number in Microsoft Excel to generate allocation sequences for each participant in the intervention and control groups, with allocation concealment by sequential numbering, representing the participant’s turn in joining the study by the principal investigator. Participants in the intervention group were identified electronically one week before the next refill schedule or follow-up visit.

### 3.2 Study participants

Between July and September 2021, 316 patients visiting the endocrinology and CDC clinics were screened for eligibility, and 281 eligible patients for inclusion were invited to participate in the study. Only 239 patients agreed to sign the consent form and randomised to either the pharmacy intervention group (120 patients) or the control group (119 patients).

In the intervention group, 15 participants were excluded at the first visit either because they refused to participate in the MTM program, or not have baseline laboratory test results or it was not done when ordered at recruitment. At the end of the study, 11 patients from the intervention group and 21 from the control group had not fulfilled the study protocol during follow-up visits or did not have exit laboratory tests and were excluded.

**Figure 1.**
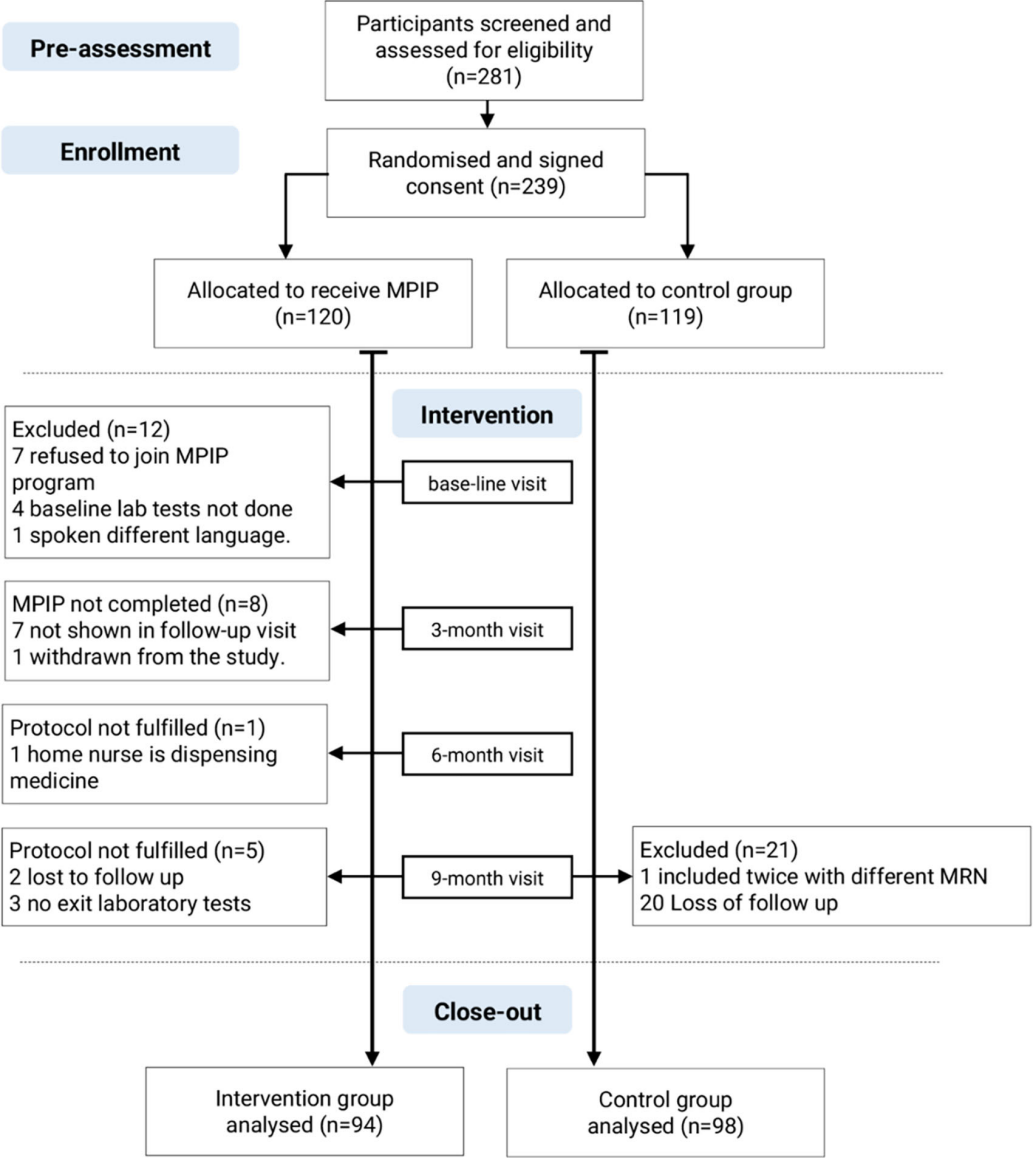
Screening, randomisation, and follow-up of the participants. MRN: Patient medical record number; MPIP: Pharmacy intervention protocol; MTM: Medication therapy management.

At the conclusion of the study after 12 months of follow-up, 94 patients in the intervention group and 98 patients in the control group were included in the analysis ( Figure 1).

### 3.3 Study procedures

At baseline, participants in both groups received medication counselling by a clinical pharmacist as part of the usual care provided in the clinic, and baseline assessment of the medication adherence was performed using a validated medication adherence questionnaire (Supplementary File 4, *Extended data* ^46^). ^16^ Participants were instructed to report any possible side effects or factors that may affect their medication adherence, and to reveal any modifications in lifestyle activities or eating habits during the study.

Participants in the intervention group joined the MPIP program on their first visit and received two multifactorial interventions: medication adherence counselling and medication therapy management, as detailed below.

*1) Medication adherence counselling*

Each participant in the intervention group received a 15- to 30-min counselling session at the initial visit and each scheduled follow-up visit which occurred every 3 months. During these sessions, the pharmacist assessed medication adherence with the adherence questionnaire and counselled the patients on the importance of medication adherence on clinical outcomes. The barriers to medication adherence were identified, discussed with the patient, and resolved as much as possible. Finally, the patient was provided with two or three months of medication supply, and the medication refill date was documented for medication adherence calculation using the medication possession ratio (MPR). ^17^^,^^18^

After each visit, each participant in the intervention group received a 15-minute follow-up phone interview within two weeks for adherence reinforcement and to address any non-compliance issues or medication side effects. Moreover, they were scheduled for their next medication refill and follow-up visits in the clinic appointment system. Appointments were scheduled at months 3, 6, and 12 from the initial visit according to the patient’s preferred date, and they received a reminder phone call three days before the next visit from the clinic call centre.

Medication adherence was evaluated using two techniques during the follow-up period: calculating the medication adherence possession ratio and the medication adherence questionnaire scores.

*A. Medication adherence possession formulas*

In this study, we used three formulas to calculate medication adherence from patients’ medication refills recorded in Cerner:

i- *Total fixed medication possession ratio (MPRt)*

A fixed medication possession ratio (MPR) formula was used to measure total adherence (composite) to all medications MPR (MPRt). ^19^^,^^20^ The MPRt was calculated for three chronic regimens including antihyperglycaemic regimen refills with any of the antihypertensive or antihyperlipidaemic regimens if the patient had any or all of them based on the medication prescription refill intervals.

For the total MPRt, no regimen-specific MPRs were measured. It was calculated by summing the days’ supply of medication per prescription, then dividing by the number of days of patient follow-up, using a fixed follow-up period of 365 days (12 months) as a constant denominator, adjusted to hospital stay if the participant was admitted during the follow-up period (Supplementary File 5, *Extended data* ^46^).

$$\text{MPRt}=\frac{\sum_{i=0}^{\mathrm{End}\;\text{of follow-up}\;\;\text{period}}\text{Days of supply}\;\mathrm{Rxi}}{\text{Days of follow-up}\;\text{period}}$$

ii- *Regimen-specific medication possession ratio (MPRs)*

Regimen-specific MPRs were calculated for all three regimens (antihyperglycaemic, antihypertensive, and antihyperlipidaemic), whether any or all were contained in the patient’s medication profile. ^21^^,^^22^

The denominator used for MPRs also covers the follow-up period of 12 months but was calculated based on the last refill date for a specific medication regimen (for any medication class) minus the first refill date for any medication in the regimen, even if this medication was in a different class from the medication used in the last refill. In addition, medications within the same regimen were considered interchangeable (even if they belonged to different medication classes).

$$\text{MPRs}=\frac{\sum \text{Days of supply of medication in}\;a\;\text{regimen}}{\text{Last refill date}-\text{First refill date}\;\left(\text{for specific regimen}\right)}$$

Of note, for both types of MPR (MPRt and regimen-specific MPRs), if the patient had refilled medication prior to the date of joining the study, MPR was calculated from the date of signing the consent, excluding the supply from the last prescription or medication refill and was capped at 1, and medication adherence (either MPRt or MPRs) was calculated after 12 months of follow-up. ^17^

The standard adherence threshold of 0.80 MPR was used as a lower bound for mediation adherence, ^20^ with MPR = 1 indicating perfect adherence, MPR < 0.80 indicating patient non-adherence, and MPR= 0 indicating no adherence. The patients’ medication refills for each medication class were recorded by Cerner (including records from private and governmental pharmacies) at each follow-up visit.

iii- *Regimen non-persistence possession ratio (RNP)*

In addition to the MPR calculation, we estimated the regimen non-persistence (RNP) or days without medication for each regimen (diabetic, hypertensive, and antihyperlipidaemic regimens) the patient had in the study, ^23^ calculated as the total number of days the patient did not receive one or more medications for any of the patient’s chronic regimen divided by the total follow-up days.

$$\text{RNP}=\frac{\sum \text{Days of supply of medication in}\;a\;\text{regimen}}{\text{Days of follow-up}\;\text{period}}$$

*B. Medication adherence questionnaire*

A validated medication adherence questionnaire translated into Arabic, showing sufficient reliability with adequate internal consistency, was used initially to measure medication adherence at baseline for all study participants, and subsequently used at each follow-up visit in the intervention group and again at the study exit (Supplementary File 4 ^46^). ^16^

The questionnaire contained four medication adherence questions from the validated Arabic version of the tool: 1) “Do you ever forget to take your medicine?” and 2) “Are you careless at times about taking your medicine?”, 3) “When you feel better do you sometimes stop taking your medicine??”, 4) “Sometimes if you feel worse when you take the medicine, do you stop taking it?”.

The total score of the medication adherence questionnaire included the first four questions with scores ranging from 0 and 4, with “Yes” having score = 1 and “No” = 0. Score improvements correspond to increased levels of adherence with higher scores implying greater levels of adherence. Patient adherence scores were classified as low adherence (score from 3 to 4), moderate adherence (score from 1 to 2), and high adherence (score = 0, with no answers with yes). ^24^

*2) Meditation therapy management (MTM) program*

As a new component of the MPIP, the MTM program was made available to the clinic’s ambulatory service for the first time, and the intervention group received the following interventions as part of the program:

*A. Medication counselling and reconciliation*

MTM was effectuated during the counselling session for 15–30 min at the time of dispensing medication. Each patient interview implemented the five criteria for MTM, including medication review to optimise patient treatment as per the latest treatment guidelines, assessment of the possibility of deprescribing, evaluation of any prescribed non-formulary drugs, patient counselling, and provision of printed medication educational materials, particularly for new, high alert, and narrow therapeutic index medications from an evidence-based drug reference to ensure the safe and proper use of these drugs (Lexicomp Online).

Moreover, the counselling session included counselling on potential drug-drug or drug-food interactions and the proper use of any device or over-the-counter (OTC) medication the patient was using. Any detailed disease information or drug modifications required were discussed and resolved by the treating physician.

Medication reconciliation and chart review were carried out through the Cerner local network, integrated with all governmental hospitals, ambulatory clinics, and private institutes in the Emirates of Abu Dhabi in the UAE.

The number and type of pharmacy clinical interventions, in addition to antihyperglycaemic deprescribing, were analysed and compared between the two groups at the end of the follow-up period.

*B. Patient medication booklet*

A pocket-sized patient-specific medication booklet was provided to each participant in the intervention group (Supplementary File 6 ^46^). This initiative in the MPIP aims to promote medication adherence in elderly patients and those with low digital skills. Moreover, the booklet helps to track OTC drugs or herbal medications bought from community pharmacies and enables patients as well as community pharmacists to fill in the name of the drug and the dose by hand, which are then manually added to the patient’s medical record in Cerner at the next MPIP visit.

During the counselling session, the counselling pharmacist attached the prescribed medication labels to the booklet, which ensured that the booklet was filled with an updated list of all prescribed medications, medication doses, allergies, next scheduled refill, and any OTC drugs the patient was currently taking.

Patients were advised to bring the medication booklet to each follow-up visit to update the next refill schedule or any changes in their profiles. Moreover, it has been used as a reference during Cerner downtime or when patients’ medical records from other private facilities were not available.

*C. SEHA mobile application*

Despite the fact that the SEHA mobile application ^25^ is accessible to all patients for download and usage, the patients often do not know how to use its features. As a new initiative in the MTM program, participants in the intervention group received face-to-face educational sessions for 5 minutes on the use of the SEHA mobile application, how to read the clinical information and laboratory results, extract their medication profile, add OTC medications to their medical record, and read notifications for medication approval from insurance as well as for subsequent medication refills.

The SEHA mobile application provides real-time individualised feedback, including patient access to Cerner, laboratory test results, radiology reports, medications, allergies, immunisation records, and health maintenance history for patient visits occurring in SEHA facilities. Moreover, it enables patients to request or reschedule appointments or reschedule them, and search for a healthcare facility. The application sends a short message service (SMS) to the registered mobile number for appointments, with reminder notifications to remain keep the patient informed about future or follow-up appointments.

At the end of the study, we used the SEHA mobile and booklet use questionnaire to record the number of participants in each group using the application on their mobile device and the extent of application utilisation (Supplementary File 7 ^46^). The results were correlated with medication adherence to measure the effect of online technology on medication management.

### 3.4 Study outcomes

The main measured outcomes in the study were: 1) change in MPRt for all regimens, 2) difference in self-reported adherence questionnaire scores between the intervention and control groups, and 3) number and type of clinical interventions between the two groups.

In addition, as secondary outcomes, we examined 1) the difference in MPRs and RNP for antihyperglycaemic, antihypertensive, and antihyperlipidaemic regimens between the two groups, and 2) the change in mobile application and patient medication booklet usage, and whether there were associations with increased patient adherence in both study groups.

### 3.5 Statistical analysis

We calculated the MPRt for the first 50 participants; the baseline MPRt was 0.93 ± 0.09 (mean ± SD). Using the Giga sample size online calculator, ^26^ we determined that a target of 82 participants per group would provide a power of 80% with an alpha level of 5%, for a minimum detectable effect of 0.035 between both groups. A total sample size of 180 was calculated, assuming a dropout rate of 10% in both groups.

Demographic and clinical data presented as categorical variables were analysed using the chi-square test, and the student’s t-test was used for continuous variables. T-test was also used to estimate the mean differences and confidence intervals for the MPR. Moreover, Cox proportional hazard analysis was utilised to compare regimen non-persistence in antihyperglycaemic, antihypertensive, and antihyperlipidaemic medications in the intervention group compared with the control group.

P values were considered significant at 0.05, with confidence intervals of 95%. Data were analysed blindly by group allocation using the IBM SPSS Statistics (RRID:SCR_019096), version 26. ^27^

Due to the lack of robust evidence to support the threshold of 0.80 for MPR to classify patients with medication adherence and MPR above the threshold as adherent to medication, we calculated regimen-specific MPRs to confirm adherence during the follow-up period, moreover, we calculated the mean difference between the intervention and control groups for MPRs for participants who achieved control in each clinical parameter adjusted to age and sex.

## 4. Results

### 4.1 Demographic data

The patients’ demographic data characteristics are presented in Table 1. ^44^ In both groups, the patient’s mean age was approximately 60 years, and 60% of them were female, with a mean body mass index (BMI) of 31. There were no significant differences in clinical baseline characteristics between the treatment groups.

**Table 1.** Characteristics of study participants at baseline.

Patients’ Characteristics	Intervention	Control	P Value
	n=94	n=98	
**Demographic**			
Age - year	59.6 ±11.7	61.8 ±13.9	0.89
BMI	30.5 (±8.6)	31.3 (±7.2)	0.95
Gender – female	67(72.8)	58 (69.2)	0.44
Marital status – married	75 (79.8)	76 (77.6)	0.72
HbA1c	7.4 ± 0.14	7.7 ± 0.13	0.54
Systolic blood pressure	136.2 ± 1.4	140.3 ± 1.1	0.77
Diastolic blood pressure	85.6 ± 0.97	87.6 ± 1.0	0.85
LDL cholesterol	3 ± 0.13	3.2 ± 0.11	0.53
Cardiovascular disease	4 (4.2)	2 (2)	0.36
eGFR below 60	11 (11.7)	15 (15.3)	0.22

Data are M ± SD or n (%).

BMI: Body mass index; (kg/m ^2^); eGFR: estimated glomerular filtration rate (mL/min/1.73 m ^2^); HbA1c: Glycated haemoglobin A1c (%, NGSP); LDL: Low-density lipoprotein (mmol/L); Systolic/diastolic blood pressure (mmHg).

### 4.2 Medication adherence

*A. Medication adherence possession data*

A trend towards overall medication adherence and total (composite) medication possession ratio (MPRt) improvement after applying MPIP in the intervention group was identified from the first follow-up visit after 3 months and the difference between groups persisted at 6, 9 and 12 months. Participants in the MPIP showed significant improvement in MPRt at 9 and 12 months of mean 0.95 ± 0.1 and 0.95 ± 0.09, compared to 0.93 ± 0.09 and 0.92 ± 0.09 in the control group respectively, with mean difference of 0.03 (95%, CI -0.01–0.05), P = 0.04, and 0.03 (95%, CI 0.01–0.06), P = 0.02, respectively ( Table 2).

**Table 2.** Medication possession ratio calculation.

Medication possession ratio	Intervention	Control	Mean difference (95% CI)	P Value
	n=94	n=98		
Follow-up time, month	11.8 ± 1.1	12.1 ± 0.76	-0.34 (-0.6- -0.08)	<0.01 *
**Total MPR for all medication**				
3 months MPRt	0.93 ± 0.09	0.92 ± 0.09	0.01 (-0.01–0.04)	0.18
6 months MPRt	0.94 ± 0.1	0.93 ± 0.09	0.01 (-0.01–0.04)	0.15
9 months MPRt	0.95 ± 0.1	0.93 ± 0.09	0.03 (-0.01–0.05)	0.04 *
12 months MPRt	0.95 ± 0.09	0.92 ± 0.09	0.03 (0.01–0.06)	0.02 *
Medication non-persistence	0.15 ± 0.21	0.22 ± 0.23	-0.07 (-0.13– -0.01)	0.02 *
Nonadherent (MPRt <0.8)	8 (8.5)	15 (15.9)	-	0.15
**Antihyperglycaemic medication** ^£^				
Regimen-specific MPRs	0.95 ± 0.09	0.92 ± 0.09	0.03 (0.01–0.06)	0.02 *
Regimen non-persistence	0.13 ± 0.16	0.22 ± 0.23	-0.09 (-0.19–0.01)	0.04 *
Nonadherent (MPRs <0.8) ^**^	8 (8.5)	15 (15.9)	-	0.15
**Antihypertensive medication** ^£^^¥^				
Regimen-specific MPRs	0.95 ± 0.1	0.92 ± 0.08	0.04 (0.01–0.06)	<0.01 *
Regimen non-persistence	0.12 ± 0.09	0.15 ± 0.18	-0.03 (-0.09–0.04)	0.23
Nonadherent (MPRs <0.8) ^**^	8 (9.3)	13 (13.8)	-	0.34
**Antihyperlipidaemic medication** ^£^^#^				
Regimen-specific MPRs	0.96 ± 0.09	0.93 ± 0.08	0.04 (0.01–0.06)	<0.01 *
Regimen non-persistence	0.09 ± 0.1	0.13 ± 0.16	-0.04 (-0.11–0.02)	0.09
Nonadherent (MPRs <0.8) ^**^	7 (7.7)	12 (12.5)	-	0.28

Data are M ± SD or n (%);

* Significant P value <0.05;

** Chi-squared test;

^£^ At study exit;

^¥^ MPIP (n=86), control (n=94);

^#^ MPIP (n=91), control (n=96); CL: confidence interval; MPRt: Total medication possession ratio; MPRs: Regimen-specific medication possession ratio, see data 1 in *Underlying data.* ^44^

**Figure 2.**
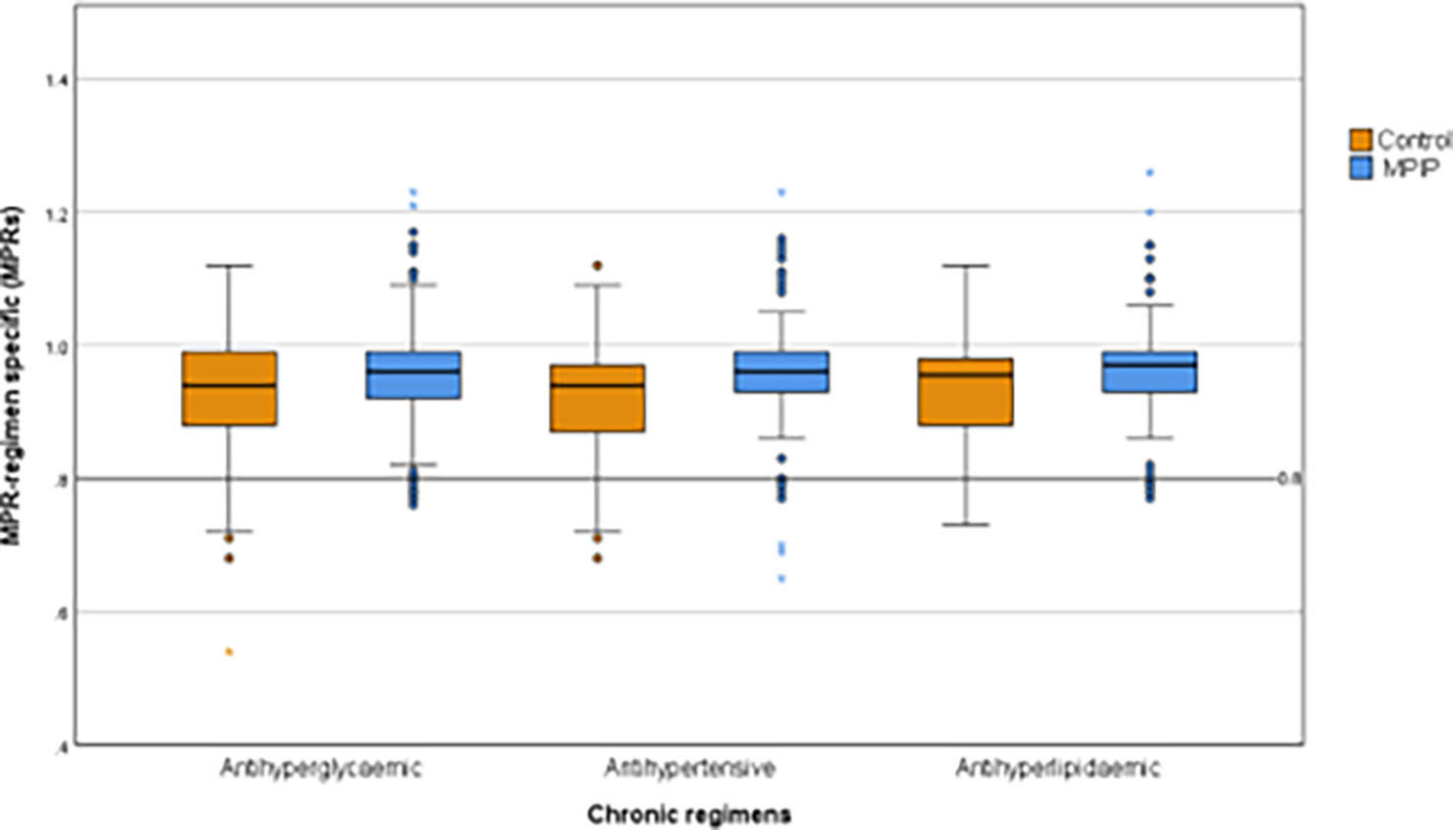
Regimen-specific medication possession ratio (MPRs) at study exit.

At the 12 months endpoint, we calculated the MPR-regimen specific (MPRs) for each chronic regimen, including antihyperglycaemic, antihypertensive, and antihyperlipidaemic medications, and we found an evident overall improvement in the MPIP group for all medication regimens ( Figure 2).

In the intervention group, the mean ± SD for MPRs was 0.95 ± 0.09 for antihyperglycaemic, 0.95 ± 0.1 for antihypertensive, and 0.96 ± 0.09 for the antihyperlipidaemic medications, compared to 0.92 ± 0.09, 0.92 ± 0.08, and 0.93 ± 0.08 in the control group respectively, with significant mean difference (95% CI) in the three regimes of 0.03 (0.01–0.06), 0.04 (0.01–0.06), and 0.04 (0.01–0.06), respectively indicating the effectiveness of the intervention protocol ( Table 2).

**Figure 3.**
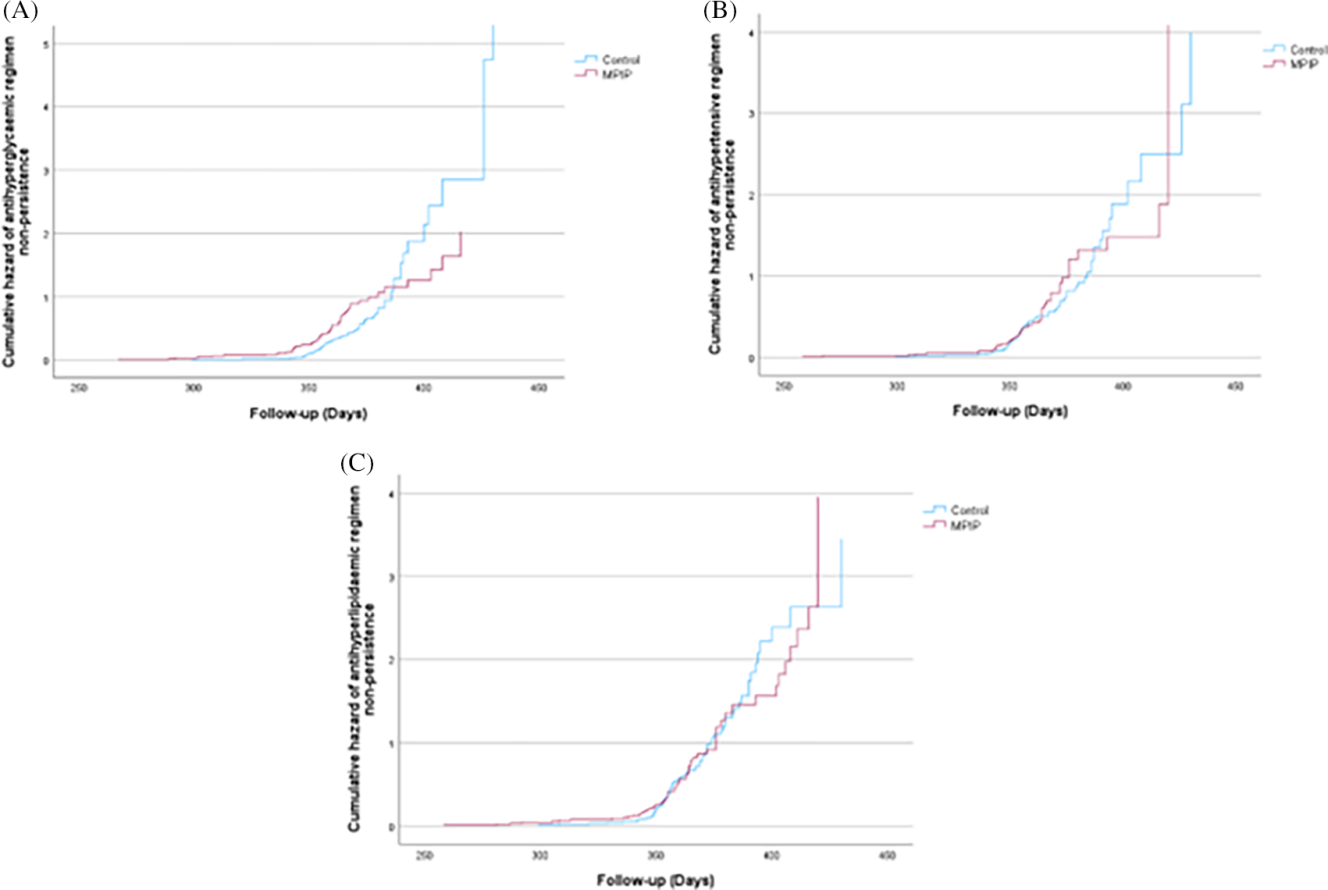
A) Cumulative hazard of antihyperglycaemic regimen non-persistence (RNP), B) antihypertensive RNP and C) antihyperlipidaemic RNP.

Moreover, compared to patients receiving MPIP, patients in the control group had more regimen non-persistence (RNP) or total number of days without medications between refills during the observation period. Participants in the intervention group had a mean (± SD) RNP in the diabetic regimen of 0.13 (± 0.16) compared to 0.22 (± 0.23) in the control group, with a significant mean difference of -0.09 (95%, CI (-0.19–0.01), P = 0.04 ( Table 2). Coherent results were observed in the RNP of the antihypertensive as well as the antihyperlipidaemic regimens; however, no statistically significant differences were detected in the mean differences or P-values or cumulative hazard function among the two regimens ( Figure 3A, B and C).

Moreover, the control group was less likely to adhere to medication regimens compared to the intervention group at the end of the follow-up period, with a higher percentage of patients with MPRt less than 0.8 compared to patients in the MPIP group. For instance, patients in the MPIP having MPRt < 0.8 and MPRs < 0.8 for the antihyperglycaemic regimen were 8 patients (8.5%) compared with 15 patients (15.9%) in the control group, despite the fact that none were statistically significant ( Table 2).

Comparable results were shown in the antihypertensive as well as the antihyperlipidaemic regimens, with MPRs < 0.8 in the intervention group = 8 patients (9.3%) and 9 patients (9.9%) compared to 13 patients (13.8%) and 12 patients (12.5%) in the control group respectively.

*B. Medication adherence questionnaire*

A tendency towards improved adherence questionnaire scores was noticeable at the end of the study, with significant improvement (P < 0.01) in the overall mean adherence score to 0.63 (± 1.1) compared to 1.1 ± 1.2 in the intervention group before commencing the study, indicating higher adherence to the prescribed medication. Moreover, at study exit, 66 participants (70.2%) in the intervention group scored zero on the questionnaire, suggesting high adherence to medication compared to 42 participants (44.6%) at baseline, and the result was significant (P < 0.01) compared to the control group (70.2% vs 48.9% in the intervention and control group, respectively) at study exit after 12 months follow-up ( Table 3).

**Table 3.** Adherence questionnaire scores.

Adherence questionnaire	Intervention (n= 94)		P-Value	Control (n=98)		P-Value	P-Value **
	Base line	Study exit		Base line	Study exit		
High adherence ^£^	42 (44.6)	66 (70.2)	<0.01 *	45 (45.9)	48 (48.9)	0.67	<0.01 *
Moderate adherence ^¥^	35 (37.2)	17 (18.1)	<0.01 *	41 (41.8)	35 (35.7)	0.38	<0.01 *
Low adherence ^#^	17 (18.1)	11 (11.7)	0.22	12 (12.2)	15 (15.3)	0.53	0.47

Data are n (%);

* Significant P value <0.05;

** P-Value: comparing intervention vs control groups at study exit;

^£^ Yes score = 0;

^¥^ Yes score from 1 to 2;

^#^ Yes score from 3 to 4.

### 4.3 Medication therapy management outcomes

*A. MTM interventions*

Pharmacists in the MPIP identified 41 drug-related problems (DRIs), implementing 36 accepted clinical interventions to optimise the dose, duration, frequency, or therapeutic substitution. In contrast, only six clinical interventions in the control group were documented, of which 5 were accepted by the prescribing physician ( Table 4). Pharmacists spent an average of 24 minutes in each counselling session, with an average of 9 minutes per intervention per patient, compared to an average of 8 minutes in counselling sessions and 6 minutes per intervention in the control group.

**Table 4.** Outcomes of medication therapy management.

MTM interventions	Intervention	Control	P Value
	n=37	n=6	
Drug-related problems	41	6	-
Accepted clinical interventions	36 (87.8)	5 (83.3)	0.76
Optimize duration	11	2	0.74
Optimize drug regimen/formulation	6	1	0.89
Optimize time of administration/frequency	9	1	0.66
Dose too high-renal	2	0	0.26
Therapeutic substitution	5	1	0.77
Drug discontinuation/duplication	3	0	0.44

Data are n (%); DRPs: Drug related problems.

At the end of the follow-up period, 26 patients in the MTM group had their medication optimised compared to only 4 in the control group; for instance, medication duration to correlate medication refill or newly added medication with the next physician following visit optimised in 11 patients, drug regimen combination and formulation optimised in 6 patients, and 9 patients had time of medication administration or frequency optimised (compared to 2, 1, and 1 in the control group, respectively).

Moreover, during patient counselling and reconciliation, two dose-related issues that were not - adjusted according to the patient’s eGFR were identified in the intervention group. Besides, five patients received medication substitution in the same or to a new therapeutic class and three patients discontinued one medication due to duplication or supratherapeutic, all from the diabetic medication regimen, to improve clinical outcomes, however, none of these results were significant ( Table 4).

At study exit, the total number of medications in the MTM group was reduced with class change in the three therapeutic classes, compared to the number of medications at study enrolment, with 249 antihyperglycaemic medications, 137 antihypertensive medications, and 119 antihyperlipidaemic medications compared with 253, 142, and 121 at baseline, respectively, with comparable medication numbers in the control group for the three classes prior to and after the study ( Table 5).

**Table 5.** Therapeutic classes characteristics for chronic regimens.

Medications in each regimen	Intervention		Control	
	Base line	Study exit	Base line	Study exit
	n=516	n=506	n=541	n=556
**Antihyperglycaemic medication**	253 (49)	249 (49.2)	274 (50.6)	280 (50.4)
Insulin	16 (6.3)	17 (6.8)	11 (4)	13 (4.6)
GLP	34 (13.4)	39 (15.7)	42 (15.3)	44 (15.7)
Metformin	84 (33.2)	84 (33.7)	86 (31.4)	83 (29.6)
Sulfonylurea	32 (12.6)	28 (11.2)	36 (13.1)	34 (12.1)
TZD	11 (4.3)	10 (4)	6 (2.2)	5 (1.9)
SGLT2	49 (19.4)	52 (20.9)	60 (21.9)	64 (22.9)
DPP4 inhibitor	27 (10.7)	19 (7.6)	33 (12)	37 (13.2)
**Antihypertensive drugs** ^¥^	142 (27.5)	138 (27.3)	152 (28.1)	163 (29.3)
Renin-angiotensin	60 (42.2)	63 (45.9)	65 (42.8)	69 (42.3)
Beta-blockers	26 (18.3)	24 (17.5)	18 (11.8)	19 (11.7)
Ca-channel BL	36 (25.3)	35 (25.5)	42 (27.6)	47 (28.8)
Diuretics	17 (11.9)	15 (10.9)	22 (14.5)	21 (12.9)
Other	3 (2.1)	1 (0.7)	5 (3.3)	7 (11.1)
**Antihyperlipidaemic drugs** ^#^	121 (23.4)	119 (23.5)	115 (21.3)	113 (20.3)
Statins	82 (67.8)	81 (68.1)	76 (66.1)	74 (65.5)
Ezetimibe	16 (13.2)	15 (12.6)	28 (24.3)	26 (23)
Omega-3	12 (9.9)	10 (8.4)	4 (3.5)	4 (3.5)
Pcsk9 inhibitors	11 (9.1)	13 (10.9)	7 (6.1)	9 (7.9)

Data are n (%);

^¥^ MPIP (n=86), control (n=94);

^#^ MPIP (n=91), control (n=95); GLP-1, glucagon-like peptide 1agonists; DPP-4, dipeptidyl peptidase 4; Pcsk9, proprotein convertase subtilisin/kexin type 9; SGLT2, sodium-glucose co-transporter-2 inhibitor; TZD, thiazolidinedione.

In antihyperglycaemic medication, the MTM group was more commonly treated with insulin (6.8% vs. 4.6%), metformin (33.7% vs. 29.6%) and less frequently with dipeptidyl peptidase 4 (DPP-4) inhibitors (7.6% vs. 13.2%) compared to the control group, which is more closely aligned with the medication recommendations of the American Diabetes Association guidelines. The usage of SGLT-2 inhibitors and GLP-1 receptor agonists was similar between the two groups ( Table 5).

Patients at risk of hypoglycaemia or adverse effects of antihyperglycaemic drugs were effectively identified using the MTM program, and the clinical pharmacist started deprescribing antihyperglycaemic drugs in collaboration with the treating physician. This included deprescribing four antihyperglycaemic medications in the sulfonylurea (32 vs. 28 at study entry and exit, respectively) class in four patients and switching them to medications with a lower risk of hypoglycaemia (two patients received GLP-1 agonists and two received SGLT2 inhibitors). Moreover, one patient in the MTM group was on mixed insulin and was switched to aspart and glargine insulin to improve nocturnal hypoglycaemia; however, the number of insulin medications increased from 16 at the study entry to 17 at the end of the follow-up period. However, both groups had similar drug profiles in the antihypertensive and antihyperlipidaemic therapeutic classes at study exit, although the patients in the MTM group were treated more frequently with Pcsk9 inhibitors compared to those in the control group (10.9% vs. 7.9%) ( Table 5).

*B. SEHA mobile application and patient medication booklet usage*

The number of patients in the intervention group who started using the mobile application for medications and clinic appointments increased from 41.5% to 45.7% in six months compared to 21.4% vs 19.3% in the control group before exiting the study (P < 0.01). Moreover, 27.6% of the patients in the intervention group started using the medication booklet provided at study recruitment and were using it during their 12 months follow-up visits compared to 1% (P < 0.01) in the control group, where the booklet was provided by a pharmacist outside the research team ( Table 6).

**Table 6.** Mobile application and medication booklet use percentages.

Variables	6 months			12 months		
	MPIP	Control	P value	MPIP	Control	P value
	(n=94)	(n=98)		(n=94)	(n=98)	
MPR <0.8	9 (9.6)	12 (12.2)	0.55	8 (8.5)	15 (15.3)	0.15
Mobile application use ^¥^	39 (41.5)	19 (19.3)	<0.01	43 (45.7)	21 (21.4)	<0.01 *
Medication booklet use ^¥^	22 (23.4)	0 (0)	<0.01	26 (27.6)	1 (1)	<0.01 *

Data are n (%);

^¥^ As one point increase (or more) in the 5-point Likert scale in the questionnaire;

* Significant P value <0.05.

## 5. Discussion

As far as we are aware, this is the first study measuring the effect of pharmacist-led interventions with multifactorial components in three chronic disease regimens together, similar studies used medication adherence possession ratios data (MPR) showed that MPR can be a reliable tool to measure adherence to one or two chronic medication regimens. ^17^^,^^18^

Moreover, this study pioneered the implementation of an MTM service with multifactorial interventions seamlessly within an existing ambulatory workflow for patients with multiple chronic conditions and taking multiple medications to improve patient outcomes, as systematic reviews demonstrated that multifactorial strategies are necessary to successfully increase adherence. ^28^^,^^29^

### 5.1 Medication adherence

Because medication adherence is a complex process, we used both objectives, through medication possession ratio, regimen non-persistence, and adherence ratios, and subjective measurements, through patient questionnaires, of patient adherence to medication. In this study, we calculated the fixed medication possession ratio (MPR) to measure adherence to dispensed medication as a valid measure of medication adherence that accounts for medication discontinuation, hospital admission, and dose change without interfering with patient behaviour. ^30^^,^^31^

To make adherence measurements more reliable in the study, we calculated three dimensions of medication adherence: total MPR (MPRt) for all regimens, MPR specific for each regimen of interest (MPRs), and medication as well as regimen non-persistence (RNP). ^18^ At the end of the 12-month follow-up period, the mean MPRt for all regimens in the intervention group was 0.95, compared to 0.92 in the control group, with a significant upward trend in the MPIP group from the third follow-visit (9 months), demonstrating the effectiveness of the intervention protocol with motivational interviewing and follow-up telephone counselling to emphasise medication adherence, which showed a sustained effect in many studies. ^32^^,^^33^

As a reliable method in measuring medication adherence, MPRs and RNP were used to validate medication adherence measurements. ^18^ The mean MPRs were 0.95 for both the diabetic and hypertension regimens in the intervention group, compared to 0.92 in the control group, and in the intervention group, the mean RNP for the antihyperglycaemic medication differed greatly from the control group (0.13 vs. 0.22), with a significant mean difference between both groups (-0.09, P = 0.04), showing the effectiveness of the multifactorial pharmacy-led intervention protocol in diabetic patients.

Comparable results were found in the antihypertensive MPRs and RNP scores, however, for the antihyperlipidaemic regimen the MPRs indicated that patients are more adherent to the antihyperlipidaemic drugs (in the MPIP and control groups) compared to other regimens in the study (0.96 and 0.93, respectively), with fewer regimen non-presistance (0.12 and 0.15, respectively). The rationale behind the greater adherence to antihyperlipidaemic drugs in the intervention group in addition to the MPIP was that 13 patients were on PCSK9 inhibitor injections at the end of the study period and 9 in the control group; these patients had scheduled appointments in the clinic for injection administration, and the approval of 6 months’ supply by the insurance company was granted after medication initiation.

Moreover, in both groups, participants were less non-adherent (MPRs < 0.8) to the antihyperlipidaemic medication (7.7% and 12.5% in the intervention and control group, respectively) compared to the antihyperglycaemic and antihypertensive medications, and the plausible reason for this was the 30 tablets packing of most antihyperlipidaemic medication compared to 28 or 56 box packing of many antihyperglycaemic and antihypertensive medications. Consequently, we modified the pharmacy-led intervention protocol to set refill dates to 28, 56, or 84, regardless of medication packing, to improve adherence to all medications in future refills in the clinic

The fixed medication possession ratio calculations were consistent with the adherence questionnaire scores as a subjective measurement of adherence: 65 (69.1%) patients reported high adherence, 18 (14.1%) patients reported moderate adherence, and 11 (11.7%) patients showed low medication adherence, compared to 42 (44.6%), 35 (37.2%), and 17 (18.1%) respectively, at study entry. On the contrary, patients in the control group achieved lower scores on the adherence questionnaire, with 48 (48.9%) highly adherent patients having zero scores, 35 (35.7%) moderate adherent patients from 1 to 2, and 15 (15.3%) low adherent patients scored from 3 to 4.

The adherence questionnaire outcomes suggested that the pharmacy-led intervention protocol was successful in improving medication adherence, indicating that the MPR is a reliable and accurate method for adherence measurement. Several studies have demonstrated that self-reported questionnaires can be a reliable strategy to measure non-adherence in patients with depression, ^34^^,^^35^ even though some studies found that few patients who scored adherent in such self-reported questionnaires were non-adherent patients. ^36^ Moreover, the MPR may overestimate adherence as it assumes that the patient is taking the medication regularly once it is in possession. In contrast, the MPR may lead to underestimation when medication is taken from a community pharmacy. ^36^ Accordingly, we used the patient’s self-reported adherence and correlated it with MPR to minimise “false-adherent” patients who have regular medication supply but fail to take them, and “false-nonadherent” patients who receive their medication from other pharmacies not linked to Cerner.

### 5.2 MPIP and Medication therapy management

The integration of a new multifactorial pharmacist-led intervention protocol (MPIP) with a medication therapy management (MTM) program improved medication adherence significantly and consistently across all treatment regimens for patients with chronic diseases, particularly in ambulatory care pharmacies, and these results corroborate the evidence from other systematic reviews and meta-analysis. ^12^^,^^13^

Pharmacists in the MPIP spared more time in the counselling sessions (24 minutes per session compared to 8 minutes in the control group), and had more time for MTM in a systematic approach utilizing Cerner for medication reconciliation, Lexi-Comp program for drug reference, patient education as well as inspection drug-drug and drug-food interactions, and scheduling patients for either phone call or visit follow-up revealed 41 DRIs (36 implemented), including two drug safety issues with unadjusted dose with low eGFR (compared to 6 in the control group), despite the insignificance between the two groups and the fewer number of antihyperglycaemic drugs in the intervention group at baseline compared to the control group, MPIP shows the effectiveness of integrating pharmacists in patients’ medication management. Studies showed that motivational interviewing, reconciliation, and deprescribing initiatives played a key role in improving medication management, and might help patients better adhere to treatment plans. ^37^^,^^38^ Moreover, it had positive outcomes in terms of drug adherence to different therapeutic regimens such as antihyperglycaemic and antihypertensive regimens. ^39^^,^^40^

MPIP allocated time in each counseling session to educate and implement two approaches integrated with the MTM program to enhance medication management and adherence outcomes: a mobile application with a patient’s medical record (SEHA mobile application), which has been shown to improve medication adherence and clinical outcomes in several studies, ^3^^,^^41^^,^^42^ and a medication booklet with the most updated medication used by the patient.

Between the two groups, we discovered meaningful variations in how frequently patients used the mobile application or their personal medication booklet, with the intervention group using them 45.7% and 27.6% more frequently by the end of the study compared to 21.4% and 1% in the control group, respectively. The increase in mobile application and patient medication booklet utilisation was consistent with fewer non-adherent patients with MPR less than 0.8 in the MPIP group compared to the control group (8.5% vs. 15.3%) ( Table 6). Thus, as evidenced in other studies, ^43^ the use of mobile applications and medication booklets can improve medication adherence in MTM programs. Lastly, these findings exhort the integration of structured, patient-centred MTM services with trained pharmacists into existing ambulatory clinical workflow in UAE.

### 5.3 Limitations and strengths

The main limitation of the MPIP is its inherent tendency to overestimate pill intake, as some patients may refill their prescriptions consistently but do not actually adhere to their medication. Moreover, the short study duration of one year with preset follow-up boundaries may underestimate compliance, resulting from occasional fluctuations in medication refills, while such errors in MPR calculation are reduced with longer periods of observation.

In addition, the mobile application was available to all participants throughout the study and was promoted by SEHA on many occasions could improve the use of the application and adherence in control patients and lead to less significant results. However, patients who underwent MPIP had higher application use and adherence rates than those who did not receive the intervention.

We did not set clinical outcomes as the primary outcome in this study, and future studies should focus on nonadherent, as well as higher-risk patients with unmet clinical goals, as adherence interventions would be more beneficial with more significant outcomes in such patients. Furthermore, the inclusion of clinical outcomes would allow for a cost-effectiveness analysis of the intervention for therapeutic. Finally, the two pharmacists were not blinded to the groups during data collection and MPR estimation. However, the objectivity of the primary outcomes and blind data analysis resolved this issue.

Regardless of these limitations, this new multifactorial pharmacy-led intervention protocol provided effective continuous monitoring and interactions to enhance patient-centred adherence and integrate readily available patient adherence data from a centralised pharmacy registry (Cerner) with patient therapeutic outcomes, which can be generalized to other ambulatory care centres. Other strengths of the study include the use of a clinical pharmacist and senior pharmacist in patient counselling, and unbiased comparisons using intention-to-treat analyses.

## 6. Conclusion

A package of multifactorial pharmacist-led interventions, including a medication therapy management program with motivational counselling, instruction on the use of a preexisting mobile application, and a patient medication booklet, modelled in a standard workflow protocol in an ambulatory pharmacy, was effective in persistently improving adherence to chronic medication, and optimising medication regimens, signifying pharmacists as invaluable assets in delivering high-quality healthcare and improving medication outcomes in diabetic patients in an ambulatory healthcare centre. Prior to implementation, larger sample sizes, longer observation periods, and cost-effectiveness analyses are required to evaluate the effectiveness of multifactorial interventions on patients’ clinical outcomes in ambulatory health services.

## Ethics and consent

This article presents one outcome of the original trial that was registered at the US National Institutes of Health ( ClinicalTrials.gov protocol registration: NCT04942119), and the original trial was approved by the Abu Dhabi Health Services Company (SEHA) Research Oversight and Ethics Committee (SEHA REC) in the United Arab Emirates on April, 2021 (approval number: SEHA-IRB-021). Written informed consent was obtained from all participants before study recruitment and they received a copy of it along with the study information sheet (Supplementary File 1, 2, and 3).

Participants had the right to withdraw consent and stop or postpone the assessment interviews or adherence questionnaires if he/she becomes upset at any time during the study period without any penalty or limitation in the usual clinical services provided. Although participants were not compensated for their extra time spent during interviews or for any inconvenience that may result from taking extra medications or blood tests every 3 months, they had prioritised access to physician’s appointments, including dietician’s visits. Furthermore, they granted direct access to our cardiologist in case of any harm from prescribing medicines or abnormal laboratory results, without any additional cost. Moreover, a 12-h counselling hotline was available during and after the study follow-up period to answer any queries or report any side effects.

## Author contributions

All authors had access to research data and contributed to the writing and review of the manuscript.

## Data Availability

Zenodo: The effects of multifactorial pharmacist-led intervention protocol on medication optimisation and adherence among patients with type 2 diabetes. https://zenodo.org/doi/10.5281/zenodo.10429457. ^44^ This project contains the following underlying data:
- Data 1.xlsx Zenodo: Supplementary Tables and Figures: “The effects of multifactorial pharmacist-led intervention protocol on medication optimisation and adherence among patients with type 2 diabetes: A randomised control trial.” https://zenodo.org/doi/10.5281/zenodo.10795195. ^45^ Zenodo: Supplementary Files: “The effects of multifactorial pharmacist-led intervention protocol on medication optimisation and adherence among patients with type 2 diabetes: A randomised control trial.” https://zenodo.org/doi/10.5281/zenodo.10795203. ^46^ Zenodo: CONSORT checklist and flowchart for ‘The effects of multifactorial pharmacist-led intervention protocol on medication optimisation and adherence among patients with type 2 diabetes: A randomised control trial’. https://zenodo.org/doi/10.5281/zenodo.10431915. ^47^ Data are available under the terms of the Creative Commons Attribution 4.0 International license (CC-BY 4.0).

## Software availability statement

IBM SPSS Statistics, version 26 (RRID:SCR_019096), ^27^ is available to the researchers in the study and they have access to the software under the licence provided to UAE university.

SEHA App (mobile application), is a free online tool and can be downloaded in all mobile’s platforms. ^25^

https://play.google.com/store/apps/details?id=com.linkdev.seha&pcampaignid=web_share

https://apps.apple.com/ae/developer/seha-abu-dhabi-heath-services-company/id436297693
