# Supplementary material for: The effects of multifactorial pharmacist-led intervention protocol on medication optimisation and adherence among patients with type 2 diabetes: A randomised control trial
Source: F1000Res. 2024 Sep 16;13:493. Originally published 2024 May 17. [Version 2] doi: 10.12688/f1000research.146517.2 (PMC11421608; doi:10.12688/f1000research.146517.2)
Supplement: Peer review history

# Reviewer report: Reviewer response for version 2

**DOI:** 10.5256/f1000research.171613.r324082

**Published:** 2024-09-23

**Authors:** Taichi Ochi

**Affiliations:** 1 University of Groningen Library, Groningen, Groningen, The Netherlands; 2 PharmacoTherapy, -Epidemiology & -Economics, University of Groningen Groningen Research Institute of Pharmacy, Groningen, Groningen, The Netherlands

Dear Authors,

 Thank you for your answers to my comments. No further questions on my side.

 Many thanks again for the nice opportunity to read this article.

Is the work clearly and accurately presented and does it cite the current literature?

Partly

If applicable, is the statistical analysis and its interpretation appropriate?

Yes

Are all the source data underlying the results available to ensure full reproducibility?

Yes

Is the study design appropriate and is the work technically sound?

Yes

Are the conclusions drawn adequately supported by the results?

Yes

Are sufficient details of methods and analysis provided to allow replication by others?

Yes

Reviewer Expertise:

Personalised care, metabolic disorders (type II diabetes), digital health

I confirm that I have read this submission and believe that I have an appropriate level of expertise to confirm that it is of an acceptable scientific standard.

# Reviewer report: Reviewer response for version 2

**DOI:** 10.5256/f1000research.171613.r324083

**Published:** 2024-09-17

**Authors:** Anas Khaleel

**Affiliations:** 1 University of Petra, Amman, Amman Governorate, Jordan

all comments answered properly

Is the work clearly and accurately presented and does it cite the current literature?

Yes

If applicable, is the statistical analysis and its interpretation appropriate?

Yes

Are all the source data underlying the results available to ensure full reproducibility?

Yes

Is the study design appropriate and is the work technically sound?

Yes

Are the conclusions drawn adequately supported by the results?

Yes

Are sufficient details of methods and analysis provided to allow replication by others?

Yes

Reviewer Expertise:

clinical pharmacy, community and hospital pharmacy research , public health, medical genetics and pharmacogenetics

I confirm that I have read this submission and believe that I have an appropriate level of expertise to confirm that it is of an acceptable scientific standard.

# Reviewer report: Reviewer response for version 1

**DOI:** 10.5256/f1000research.160610.r305397

**Published:** 2024-07-25

**Authors:** Anas Khaleel

**Affiliations:** 1 University of Petra, Amman, Amman Governorate, Jordan

Dear authors

 Please see comments below

**Introduction**

 The introduction provides relevant statistics, which can be strengthened by incorporating primary data or pilot study findings to support the problem statement.

 The introduction could benefit from grounding the study in a theoretical framework related to behaviour change or medication adherence.

 Expand on the theoretical framework underpinning the study to strengthen the rationale.

- Strengthen the link between the problem statement and the proposed intervention.

Although the Emirates of Abu Dhabi

 Emirate no S

- Expand on the description of the intervention components in the MPIP program.

Randomization

 What type of randomization was used in this RCT?

- **Justification for sample size missing:** The rationale behind the sample size (239 patients) is not provided. Is there sample size calculation ? please state how sample size were calculated
- **Potential selection bias:** It's unclear how researchers addressed it during recruitment.

OTC abbreviations are not spelled out throughout the manuscript

  SEHA mobile and booklet use questionnaire (do these booklets and apps have Arabic versions?)

- T-test was also used to estimate the mean differences and confidence intervals for the MPR…

Did you use paired or independent t-tests?

***Results***

**Figure 2**



 Image not clear

**Figure 2**

 Please provide high-resolution picture

***Discussion***

- Expand on the discussion of clinical outcomes, even if they were not the primary focus of the study.
- Strengthen the comparison of the study's results to existing literature to highlight the study's contribution to the field.
- Conduct a more comprehensive analysis of potential confounding factors.
- Provide clear and actionable recommendations for clinical practice based on the study's findings.

In conclusion please provide an insight about the significant role of pharmacist in healthcare team

Is the work clearly and accurately presented and does it cite the current literature?

Yes

If applicable, is the statistical analysis and its interpretation appropriate?

Yes

Are all the source data underlying the results available to ensure full reproducibility?

Yes

Is the study design appropriate and is the work technically sound?

Yes

Are the conclusions drawn adequately supported by the results?

Yes

Are sufficient details of methods and analysis provided to allow replication by others?

Yes

Reviewer Expertise:

clinical pharmacy, community and hospital pharmacy research , public health, medical genetics and pharmacogenetics

I confirm that I have read this submission and believe that I have an appropriate level of expertise to confirm that it is of an acceptable scientific standard, however I have significant reservations, as outlined above.

# Response

**Published:** 2024-09-07

**Authors:** Marwan El-Deyarbi

**Affiliations:** Pharmacology, United Arab Emirates University, Al Ain, Abu Dhabi, United Arab Emirates

Thank you for your time and efforts to review our research work. Please note that all of your valuable comments have been addressed carefully and changes have been made in the manuscript accordingly.

 Kind regards

 Reviewer comments:  

1. **Introduction**:  

- **The introduction provides relevant statistics, which can be strengthened by incorporating primary data or pilot study findings to support the problem statement. The introduction could benefit from grounding the study in a theoretical framework related to behaviour change or medication adherence.**
** Expand on the theoretical framework underpinning the study to strengthen the rationale.**

Thank you for your valuable comments. The results of a systematic review study and another meta-analysis study have been added in the introduction in Line 24 and Line 26, Page 4 (PDF File).

-  
**Strengthen the link between the problem statement and the proposed intervention.**
  - Thank you for the important comment. Paragraphs number 8 and 9 (on Page 4 in [PDF File]), have been merged to strengthen the link between the problem and the proposed intervention.

1. **Expand on the description of the intervention components in the MPIP program.**

Thank you for your comment. The MPIP program has been listed as:1) Medication adherence counselling including measuring medication adherence by medication adherence possession formulas and validated medication adherence questionnaire, and 2) a Meditation therapy management (MTM) program, by implementing the five criteria for MTM, including medication review to optimise patient treatment as per the

 latest treatment guidelines, assessment of the possibility of deprescribing, evaluation of any prescribed non-formulary drugs, patient counselling, and provision of printed medication educational materials through:

 A) Medication counselling and reconciliation,

 B) Patient medication booklet, and

 C) SEHA mobile application.

1. **Randomization: **
  - **What type of randomization was used in this RCT?**

Thank you for your comment. Simple randomisation was performed using a computer-generated random number in Microsoft Excel to generate allocation sequences. The word “simple” has been added to the manuscript (Line 25, Page 5 [PDF File]).

- **Justification for sample size missing: The rationale behind the sample size (239 patients) is not provided. Is there sample size calculation ? please state how sample size were calculated**

Thank you for your comment. The sample size has been detailed in the Statistical analysis section 3.5.  In the study, we used Giga sample size online calculator to detect a minimum detectable difference effect of 0.035 between both groups with an MPRt baseline mean of 0.93, and a minimum total sample size of 180 was determined. However, a larger number of participants (239 participant) was recruited for more significant differences between groups and to account for any dropout during the study.

  

- **Potential selection bias: It's unclear how researchers addressed it during recruitment.**
  - Thank you for the insightful remark. The potential of selection bias has been minimized during recruitment with allocation concealment by sequential numbering, representing the participant’s turn in joining the study by the principal investigator (Line 26, Page 5 [PDF File]).

 

- **OTC abbreviations are not spelled out throughout the manuscript**

Thank you for your comment. Over-the-counter (OTC) has been added to the manuscript (Line 24, Page 8 [PDF File]).

1. **SEHA mobile and booklet use questionnaire (do these booklets and apps have Arabic versions?)** Thank you for your comment. Both the patient’s medication booklet (in Supplementary Files,  Supplementary File 6), and SEHA mobile application have both an Arabic and English interface. (A link for the SEHA application in both Google and Apple stores has been added to the manuscript)
2. **T-test was also used to estimate the mean differences and confidence intervals for the MPR…Did you use paired or independent t-tests?**

Thank you for your comment. For mean differences and confidence intervals, independent t-tests were used in SPSS (version 26).

1. ***Results***
  See Figure 2 linked here: *https://f1000research.s3.amazonaws.com/linked/674183.146517.pdf*

**Image not clear Figure 2. Please provide high-resolution picture**

Thank you for your valuable comment. All figures and tables are available in high resolution in the “Extended data” section (Line 26, Page 17 [PDF File]).

 Zenodo: Supplementary Tables and Figures: “The effects of multifactorial pharmacist-led intervention protocol on medication optimisation and adherence among patients with type 2 diabetes: A randomised control trial.” https://zenodo.org/doi/10.5281/zenodo.10795195.45

  

1. **Discussion**
**Expand on the discussion of clinical outcomes, even if they were not the primary focus of the study.**

Thank you for your valuable comment. The clinical outcomes will be published in a separate manuscript, and a statement of publishing one outcome from the original trial is available in the manuscript (Line 11, Page 5 [PDF File]).              

  

- **Strengthen the comparison of the study's results to existing literature to highlight the study's contribution to the field.**

Thank you for your comment. A comparison with other studies has been added in the discussion section (Line 3 and 5, Page 15 [PDF File]).     

  

- **Conduct a more comprehensive analysis of potential confounding factors.**

Thank you for this important comment. Another manuscript (under review for publication) is discussing the factors affecting medication adherence including socio-demographic variables, medication-related barriers, healthcare system-related barriers, financial or logistical barriers, and perception and social-specific confounding factors.

  

- **Provide clear and actionable recommendations for clinical practice based on the study's findings.**

Thank you for your comment. A recommendation has been added at the end of the discussion section (Line 24, Page 16 [PDF File]).

  

- **In conclusion please provide an insight into the significant role of pharmacists in healthcare team**

Thank you for your valuable comment. A sentence pointing out the importance of the pharmacist has been added to the conclusion ( Section 6, Page 16 [PDF File]).

# Reviewer report: Reviewer response for version 1

**DOI:** 10.5256/f1000research.160610.r284388

**Published:** 2024-06-24

**Authors:** Taichi Ochi

**Affiliations:** 1 University of Groningen Library, Groningen, Groningen, The Netherlands; 2 PharmacoTherapy, -Epidemiology & -Economics, University of Groningen Groningen Research Institute of Pharmacy, Groningen, Groningen, The Netherlands

Dear authors,

 Thank you for your article. It was insightful to read about the examination of the new MPIP program in the UAE to improve patient outcomes of patients with type II diabetes. After reading your article, I have the following points that I would like to highlight for further revision.

- The figures provided for diabetes prevalence could be updated. Citing the recent IDF Diabetes Atlas reports would shed light on more recent numbers. Furthermore, as the study focuses on patients with T2D, I would suggest to focus on the figures for T2D, not diabetes overall (if possible for the UAE). 
- When comparing the medications in each regimen (Table 5), the baseline between the two groups was different. This could be partly explained as more participants were in the control. However, no further discussion on this is detailed. Was this something that was considered to impact the analysis?
- An issue faced by more elderly patients is the number of medications prescribed to address different medical conditions. While the methods mentioned that the counselling would discuss potential DDIs/DFIs, no information regarding the patient's other conditions was detailed. Would it be possible to include this information (i.e. Table 1)?
- The benefits of the MPIP on mobile application/medication booklet use are very clear. However, what is considered as use was not defined. Is opening the app once between the visitation periods considered use? Or a weekly/monthly checking of medical information? Or solely for booking an appointment?
  If you could clarify this further, that would be greatly appreciated. (the citation to the app page did not work for me but found it on the Google Playstore)
- For grammar, I would like to mention that there are many run-on sentences throughout the paper. This can lead readers to lose track of the aim of the sentence. For revision, I would suggest simplifying sentences by limiting the use of 'and' to connect points. 

In addition, I have a couple of minor points that I would like to highlight.

- For Table 1, if you could specify the unit of measure for HbA1c, blood pressures, LDL cholesterol, that would be appreciated (for clarity). 
- For the cited (supplementary) files (citation 44 - 47), you could add the DOI of the Zenodo link to make it easier to access for readers (some other articles are also missing DOI/linkage)

Many thanks again for your article and consideration.

Is the work clearly and accurately presented and does it cite the current literature?

Partly

If applicable, is the statistical analysis and its interpretation appropriate?

Yes

Are all the source data underlying the results available to ensure full reproducibility?

Yes

Is the study design appropriate and is the work technically sound?

Yes

Are the conclusions drawn adequately supported by the results?

Yes

Are sufficient details of methods and analysis provided to allow replication by others?

Yes

Reviewer Expertise:

Personalised care, metabolic disorders (type II diabetes), digital health

I confirm that I have read this submission and believe that I have an appropriate level of expertise to confirm that it is of an acceptable scientific standard, however I have significant reservations, as outlined above.

# Response

**Published:** 2024-09-07

**Authors:** Marwan El-Deyarbi

**Affiliations:** Pharmacology, United Arab Emirates University, Al Ain, Abu Dhabi, United Arab Emirates

**Dear Respected Editor and Reviewer,**

 Thank you for your time and efforts to review our research work. Please note that all of your valuable comments have been addressed carefully and changes have been made in the manuscript accordingly.

 Kind regards

**Reviewer comments: **
** **

- **The figures provided for diabetes prevalence could be updated. Citing the recent IDF Diabetes Atlas reports would shed light on more recent numbers. Furthermore, as the study focuses on patients with T2D, I would suggest to focus on the figures for T2D, not diabetes overall (if possible for the UAE**). 

Thank you for your comment. The updated IDF Diabetes Atlas data has been added in the introduction section(Line 2, Page 4 [PDF File]).

- **When comparing the medications in each regimen (Table 5), the baseline between the two groups was different. This could be partly explained as more participants were in the control. However, no further discussion on this is detailed. Was this something that was considered to impact the analysis?**

Thank you for your comment. I agree with you that there was a difference in medication regimen between both groups at baseline; however, the HbA1c levels at baseline were 7.4 and 7.6 for the intervention and control, respectively, with 6.3% of participants in the intervention group receiving insulin as part of their diabetic regimen compared to 4% of participants in the control group (Other chronic medication’s regimens were well balanced among the control and intervention groups at enrollment).

 Moreover, it didn’t impact the analysis as we measured medication adherence possession data based on the actual number of medications in each patient and in the MTM study we presented the percentage change from baseline to study exit. In addition, the higher number of antihyperglycaemic drugs in the control at baseline may show that antihyperglycaemic deprescribing in the MTM program is more efficient and the below comment has been added in the discussion (Line 11, page 16 [PDF File])

*Added: “ and the fewer number of antihyperglycaemic drugs in the intervention group at baseline compared to control group.”*

- **An issue faced by more elderly patients is the number of medications prescribed to address different medical conditions. While the methods mentioned that the counselling would discuss potential DDIs/DFIs, no information regarding the patient's other conditions was detailed. Would it be possible to include this information (i.e. Table 1)?**

Thank you for valuable comment. Number of patients with cardiovascular disease and chronic kidney disease (eGFR below 60) has been added to Table 1

  

- **The benefits of the MPIP on mobile application/medication booklet use are very clear. However, what is considered as use was not defined. Is opening the app once between the visitation periods considered use? Or a weekly/monthly checking of medical information? Or solely for booking an appointment?**
** If you could clarify this further, that would be greatly appreciated. (the citation to the app page did not work for me but found it on the Google Playstore)**
  1. Thank you for the insightful remark. The increase of use has been defined as one point increase (or more) in the 5-point Likert scale in question 1 in the SEHA mobile and booklet use questionnaire table ( supplementary File **7** in the supplementary document Link ” https://zenodo.org/doi/10.5281/zenodo.10795203 ^46^” ) at each follow-up visit or at study exit for any purpose either for appointments, medication or lab review ( the below clarification added in the footnote of Table 6 (PDF File)
** **“ As one point increase (or more) in the 5-point Likert scale in the questionnaire.”
  2. A link for the SEHA application in both Google and Apple stores has been added to the manuscript.
- **For grammar, I would like to mention that there are many run-on sentences throughout the paper. This can lead readers to lose track of the aim of the sentence. For revision, I would suggest simplifying sentences by limiting the use of 'and' to connect points. **

Thank you for the important comment. In the revised version, many sentences have been connected by limiting the use several connecting words and the use of “ and” to join ideas and sentences

**In addition, I have a couple of minor points that I would like to highlight.**

- **For Table 1, if you could specify the unit of measure for HbA1c, blood pressures, LDL cholesterol, that would be appreciated (for clarity). **

The units of measure for HbA1c, blood pressure, eGFR, and  LDL cholesterol have been added in the footnote of Table 1 (PDF File).

- **For the cited (supplementary) files (citation 44 - 47), you could add the DOI of the Zenodo link to make it easier to access for readers (some other articles are also missing DOI/linkage).**

Thank you for your comment. DOI of the Zenodo for citations from 44 to 47 has been added to the revised file.
